# Test-retest reliability of adolescents’ self-reported physical activity item in two consecutive surveys

**DOI:** 10.1186/s13690-019-0335-3

**Published:** 2019-02-25

**Authors:** Kwok Ng, Riikka Hämylä, Jorma Tynjälä, Jari Villberg, Tuija Tammelin, Lasse Kannas, Sami Kokko

**Affiliations:** 1 Research Centre of Health Promotion, Faculty of Sport and Health Sciences, University of Jyvaskyla, PO Box 35, 40014 Jyväskylä, Finland; 2 Department of Physical Education and Sport Sciences, University of Limerick, Limerick, Ireland; 3 School of Educational Sciences and Psychology, University of Eastern Finland, Joensuu, Finland; 4 LIKES Research Centre for Physical Activity and Health, Jyväskylä, Finland

**Keywords:** Questionnaire design, Self-report, Adolescents, Physical activity, Epidemiologic monitoring

## Abstract

**Background:**

National monitoring of school-aged physical activity (PA) behaviours is necessary to inform policy makers. The Finnish School-aged Physical Activity (FSPA – LIITU in Finnish) is a physical activity monitoring study, collecting data from young adolescents aged 11 to 15 years through a nationally representative sample. This study included a single self-reported item question on moderate to vigorous intensity physical activity (MVPA) from the preceding seven days. The question is used widely in the WHO Collaborative Cross-National Health Behaviour in School-aged Children (HBSC) study as a measure of meeting international PA recommendations. This study evaluated the test-retest reliability of the aforementioned MVPA item in two consecutive surveys while observing gender and age categorisation differences.

**Methods:**

In this study, Finnish adolescents with mean ages of 11.5y, 13.5y and 15.5y (*n* = 2752) completed the HBSC and FSPA surveys in two 45 min class periods without a break in 2014. The HBSC survey completion mode was through pen and paper, and the FSPA study through a web-based questionnaire. The same MVPA question appeared in both surveys. Response alternatives (0–7 days per week) were grouped into four, and two categories in the analyses. Cohen’s Kappa and ICC statistics were performed to test the intra-rater test-retest reliability of the measure.

**Results:**

According to Cohen’s Kappa, there was moderate agreement through the use of four (0.503) and two (0.599) categories, however, the proportion of adolescents that met the recommended daily 60 min of MVPA was 8% lower in the FSPA study than in the HBSC study (19% vs 27%). In addition, ICC for MVPA, as continuous variable (0–7 days) had good to excellent reliability (range 0.694–0.765) for boys and girls aged 13 to 15 years, but only fair (0.565) for boys aged 11.

**Conclusions:**

This study demonstrated that single item MVPA item was considered to have acceptable reliability of the measure for monitoring purposes of 13- and 15-year old boys, and 11y-, 13- and 15y-old girls meeting the international PA recommendations. There were differences in the prevalence in daily MVPA due to survey design.

## Background

National monitoring of health behaviours is important tool for health agencies, researchers and other stakeholders allowing policy makers take note of health promotion, intermediate health, and societal health outcomes [1]. Physical activity (PA) is an important health promotion activity because physical inactivity is the fourth leading risk factor for global mortality [2]. Sufficient amounts of PA provides protection from disease, disability or injuries [3] as well as increased social interaction and improved mental and physical health [4, 5]. PA levels in adolescents require regular monitoring because rapid biological, psychological and social changes occur [6]. The WHO collaborative cross-national Health Behaviour in School-aged Children (HBSC) study and the Finnish School-aged Physical Activity (FSPA – LIITU in Finnish) study are important national monitoring tools for adolescent’s physical activity behaviours. The HBSC study is performed every quadrennial, and the SPA conducted biennially. The two surveys were completed at the same time during 2014, providing opportunities for interdisciplinary research in adolescent health.

At an international level, the HBSC study was developed to gain new insight and increase understanding of adolescent health behaviours, health, lifestyles well-being, and their social contexts in different countries [7]. At its inception there were three countries involved in 1983, in 2014 there were 45 countries in Europe and North America involved for national and international monitoring purposes. The study includes various health behaviours of young adolescents such as, eating and drinking, risk behaviours, positive health behaviours, experience of violence and injury, as well as family culture. Physical activity has been a core area of interest in the HBSC study, however items do not sufficiently cover detailed information about PA behaviours, habits and social context.

To overcome this important issue, the FSPA study was developed and acts as a national monitoring survey. Its first year of data collection was in 2014. Data were collected through cross-sectional methods and the intention for the survey is for regular monitoring every two years. As a PA specific survey, the research group behind FSPA recognises the importance of combining self-reported PA with device worn measurements of PA. Therefore, in the 2016 data collection, over 3000 adolescents used device based measures to supplement self-reported responses from the questionnaire [8]. However, in the 2014 study, as the first cycle of data collection, only self-reported questionnaires were used.

Information regarding the frequency, intensity, time and type of PA is difficult to capture in adolescents [9], particularly so, when PA can also take place in free living environments. However, self-reported PA has been used in large monitoring surveys [10]. When standardised with other surveys, results become comparable for international studies [11–13]. Although the methods have come under heavy scrutiny [14], the logistics of collecting through self-report so far, outweigh the costs and difficulties of collection through device based measurements [15, 16].

### Reliability of self-reported PA over time

There have been studies that have examined the test-retest reliability of a single item recall measure of the preceding seven days PA [17–19]. In these studies, administration of tests had gaps between one to four weeks. Intraclass Correlation Coefficients (ICC) values were considered to be excellent (ICC = 0.98) in a test-retest when the gap was one week [17], although reduced between 0.7 and 0.8 when the gap was two weeks [19]. When the gap was three weeks, ICC value was 0.82 [17] and was much lower, ranging from 0.51–0.53, when the gap was 4 weeks [18]. Findings from these studies suggest time between testing is an important factor for reporting reliability of instruments. Changes in weather and other activities during the week may change from one week to the next and this affects participation in physical activities. Therefore, responses to items do not correlate excellently [17, 18].

One possibility to reduce these potential measurement errors would be to conduct the same questions on the same day. However, it may present recency effects and cause bias to the responses. The use of cognitive overloading has been seen to remove recency effects from data collections [20]. Therefore, to conduct a test over the same day, it is important that between items, respondents have to answer many detailed questions about their own personal behaviours. This was a basic consideration for how to set up a consecutive test of reliability for the self-reported measures in PA.

### Aims

The aims of the study were threefold. One, to examine the intra-rater test-retest reliability of a self-reported seven-day recall PA measure. The second aim was to study the changes of instrument reliability by age. The third aim was to explore how reclassifications of response categories have an effect on the test-retest reliability.

## Methods

### Sample

The data collections for the HBSC and FSPA studies were conducted together between March and May 2014. The HBSC data were based on a nationally representative sample of 11-, 13- and 15-year-old Finnish adolescents. For the HBSC study, 539 of 2420 Finnish schools were selected from the Statistics Finland’s 2012 register of educational institutions. Sampling was carried out with probability proportional to size, with regional stratification and clustering. The school was the primary sampling unit. The sampling was done separately for age groups 11, 13 and 15 years old. From the sampled schools, one class per grade was selected randomly to participate in the study [21]. The response rate for the HBSC study was 85% of adolescents (*n* = 6414). The sampling and data collection were done according to the research protocol of international HBSC Study (more information about HBSC data collection can be found from Currie et al. 2014 [7].

**Fig. 1:**
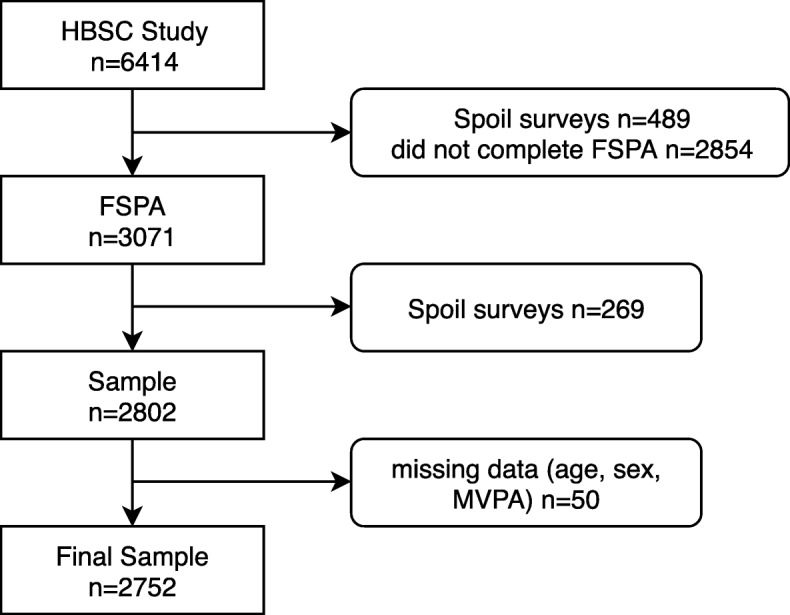
Flow chart of participants in the overall survey

As part of the HBSC protocol [7], the file had to be cleaned and the final data file consisted of 5925 respondents. In addition to the HBSC Study, over half of the respondents (*n* = 3071) took part in the FSPA study. After the cleaning data process, the sample for the SPA study consisted of 2802 respondents (Fig. 1). After removal of participants with missing values from the combined data set, the final sample used in this test-retest study was 2712 respondents (52% female, 11y – 31.8%, 13y – 33.1%, 15y – 35.1%).

For the administration of respondents who completed both surveys, teachers were present and administered them in the classrooms. Two consecutive lesson slots (2 × 45 min) were reserved to complete the two questionnaires. In addition, no recess /breaks between the lessons were permitted. Teachers were sent packages with instructions for implementing studies. During the first 45 min lesson, the adolescents completed the HBSC questionnaires in paper form. Afterwards, adolescents used a web-based questionnaire for the FSPA study. The respondents were given instructions to fill their unique codes from the HBSC questionnaire into the web-based questionnaire of FSPA. The two questionnaires were then linked with these unique codes. Researchers had no information about the respondent and the codes, ensuring all responses remain anonymous. Participation was voluntary and anonymous in the both studies.

### Instruments and variables

#### HBSC study

To conform with the national and international HBSC study protocol, there were 74 questions in the 2014 study. Questions had undergone a back translation process, with verification from within the researchers at the network. The standard physical activity question was the first question respondents encountered about the subject and was placed towards the beginning of the survey. This is important to note because respondents needed to go through the rest of the survey covering other questions related to health behaviours, including sensitive ones like romantic relationships, substance use and family cultures. With the breadth of questions, respondents were likely to experience cognitive overload, thus reducing recency recall bias for completion of the question in the FSPA questionnaire.

#### FSPA study

The survey design for the FSPA study had some distinct features from the HBSC study. The 2014 FSPA survey was a web-based questionnaire, completed in a computer classroom. The focus of the questionnaire was on physical activity behaviours. As such, the layout of the page with the question of weekly physical activity included first, an item concerning light intensity PA then, preceding seven days moderate intensity PA, and then, an average seven days moderate intensity PA item.

#### Physical activity

The moderate to vigorous physical activity (MVPA) item has been a core question in the WHO Collaborative Health Behaviour in School-aged Children (HBSC) study since 2002 [22] and was used in both HBSC and FSPA studies in 2014. An introductory text was present in both surveys, “Physical activity is any activity that increases your heart rate and makes you get out of breathe some of the time. Physical activity can be done in sports, school activities, playing with friends, or walking to school. Some examples of physical activity are running, walking briskly, roller-skating, cycling, dancing, skateboarding, swimming, downhill skiing, cross-country skiing, football, basketball and baseball.” was placed before the question; “Over the past 7 days, on how many days were you physically active for a total of at least 60 minute per day? Please add up all the time you spent in physical activity each day.” The response options were 0 days; 1; 2; 3; 4; 5; 6; 7 days.

Rarely is the full-scale used when reporting overall PA behaviours. Therefore, analyses were based on the various possible categorical approaches. For the purpose of testing the instrument, one classification was based groups of four or two categories. Four categories were defined as: 0–2 (inactive), 3–4 (slightly active), 5–6 (those who almost fulfil the recommendations), and 7 days (those who fulfil the PA recommendations). Another categorical approach was more crude: 0–6 (those who do not fulfil the recommendations) and 7 days (those who fulfil the recommendations).

#### Type of statistical analyses

Statistical analyses were conducted with age categories and gender stratified. Response shifts from one survey to the next were calculated from the reported number of days from the HBSC to the number of days reported in the FSPA study. Negative and positive shifts of the same amount were combined to give an indication of the magnitude away from the exact same result. Descriptive statistics as well as chi-square tests of independence were performed on gender and age categories for these response shifts.

The reliability of the instrument was tested with Cohen’s Kappa statistics. Kappa range of 0.00–0.20 is quantified as poor strength of agreement, 0.21–0.40 can be seen as fair agreement, 0.41–0.60 is moderate, 0.61–0.80 good and 0.81–1.00 very good strength of agreement for categorical variables [23]. Kappa statistics were used with MVPA item classified into four and two categories. In addition to Kappa statistics, an intra-rater reliability with absolute agreement with ICC was used to evaluate the reliability of the MVPA item as a continuous variable. ICC values between 0.60–0.74 were considered as good and over 0.75 were considered excellent [24].

Additional analyses were performed to allow some compensation in the responses between surveys by shifting +/− 1 day. The data were analysed at first, for the full sample and then separately for boys and girls in different age groups. The analyses performed using IBM SPSS Statistics 22.0.

## Results

**Fig. 2:**
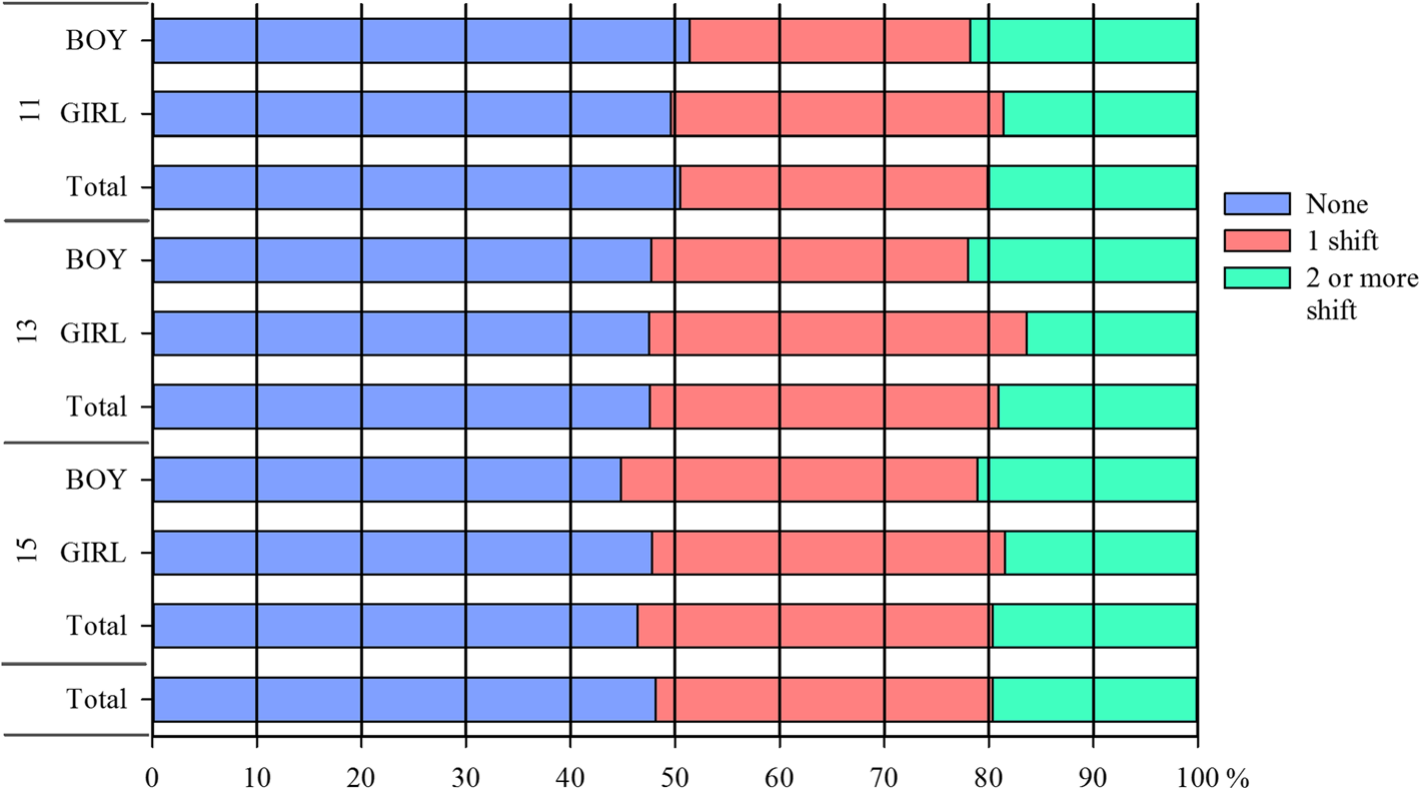
Percentages of test-retest response shifts of physical activity (days) in HBSC and SPA 2014 surveys

The proportion of exactly the same responses between the HBSC and FSPA studies were just under half (11y – 50.5%, 13y – 47.6%, 15y – 46.4%) (Fig. 2). Tests of differences between age categories (*p* = 0.263) was not statistically significant, however more girls (47.9%) significantly reported no shift (in exact responses) than boys (46.5%) (*p* = 0.024). The proportion of respondents who responded exactly the same response after allowing for a shift in +/− 1 day was greater than zero day shift and similar across the age groups (11y – 80%, 13y – 81%, 15y – 81%).

The Kappa values and ICC were stratified by gender and age. These are shown in Table 1. According to the interpretations of Cohen’s Kappa by Altman [23], there were moderate agreement levels when there were four (0.50) and two (0.60) categories.

**Table 1:** Kappa and ICC for MVPA item in HBSC and FSPA questionnaires by gender and age

		MVPA 4 categories Kappa	MVPA 2 categories Kappa	MVPA continuous ICC
11-year olds				
Boys		0.439	0.539	0.565
Girls		0.505	0.579	0.665
13 year olds				
Boys		0.465	0.547	0.694
Girls		0.507	0.621	0.745
15 year olds				
Boys		0.525	0.615	0.765
Girls		0.492	0.586	0.743
Total		0.503	0.599	0.720

Through the use of Cichetti’s [24] interpretation of ICC values there was good reliability among boys and girls in age groups 13y- and 15y-olds with ranges from 0.69 to 0.77. In addition, 11y-old girls (0.67) had good ICC values, but 11y-old boys did not.

After collapsing the responses into four categories, there were no remarkable differences between 11y-, 13y-, and 15y-old age groups (62, 61, 63%, respectively) or between boys (60%) and girls (64%). However, when two categories were analysed, more 15-year-olds chose the same answer in both questionnaires than younger respondents (11y – 80%, 13y – 83%, 15y – 90%) and more girls (87%) reported the same in both surveys than boys (81%).

### Reporting PA recommendations

In Table 2, more respondents reported to have met the PA recommendations in the HBSC study (27%) than the FSPA study (19%). The patterns of gender differences in PA and decline of PA as age increased remained the same in both surveys. In addition, there was an increasing proportion of adolescents who reported no days of MVPA as age increased.

**Table 2:** Proportion (%) of adolescents of 0–7 days of moderate to vigorous physical activity among 11-, 13- and 15-year-old boys and girls according to the HBSC and FSPA data

			0 days	1	2	3	4	5	6	7 days	Total (n)
11-year-olds	Boys	HBSC	1	2	3	9	9	12	14	50	441
		FSPA	1	3	6	9	13	15	19	36	441
	Girls	HBSC	1	4	2	10	16	16	21	30	466
		FSPA	1	3	5	17	16	15	18	25	466
13-year-olds	Boys	HBSC	3	3	6	12	13	18	12	32	450
		FSPA	4	7	8	16	14	18	12	22	450
	Girls	HBSC	1	3	7	14	18	20	14	22	462
		FSPA	2	4	10	17	21	19	13	16	462
15-year-olds	Boys	HBSC	3	6	10	16	20	17	10	19	438
		FSPA	5	9	13	18	20	16	9	11	438
	Girls	HBSC	3	6	13	20	20	16	10	13	495
		FSPA	4	9	17	19	17	16	9	9	495
Total		HBSC	2	4	7	14	16	17	14	27	2752
		FSPA	3	6	10	16	17	16	13	19	2752

## Discussion

According to the results of this study, almost half of Finnish adolescents recalled exactly the same amount of PA in both surveys. The prevalence in daily MVPA from the HBSC survey was higher than the FSPA survey. From this sample in this study, just over a quarter of adolescents (27%) who completed the HBSC survey, reported to take part in at least 60 min of MVPA every day. This was higher than the one in five (19%) that reported the same amount of PA in the FSPA study. These differences were examined through test-retest statistics, whereby there was moderate agreement when the scale was reduced to four or two categories. Survey designs considerations were used to explain the overall findings.

Despite completion of the test on the same day, less than half of the adolescents were able to recall exactly the same response between the two surveys. Most studies concerning reliability of self-reported PA were repeated within a few hours to a few weeks [6, 25] or even up to three months [16]. Chinapaw and colleagues [26] recommended that adequate time between test and re-test is more than one day but shorter than two weeks when measuring PA through this measure. Time between the two tests has an influence on reliability [6, 27]. Bobakova and colleagues [18] suggested that if the time between the two questionnaires is short, the respondents might remember their answers. However, with less than half reporting exactly the same response, this would suggest that there were no recency effects from this type of study design. The adolescents were cognitively challenged to respond to over 50 other personal questions in the pen and paper survey after stating the number of days they participated in PA. This cognitive overloading exercise is likely to remove the potential recency effects [28].

The problem with conducting test-retest reliability studies on behavioural patterns is individual behaviour has variations in itself [16]. Participation in PA varies daily [29] and may be influence by many factors including seasonal aspects, weather conditions, school and family activities [18, 30, 31]. As such, this may lead to lower reliability scores [32]. In our study, the time gap between the two questionnaires was almost non-existent, and there were no changes in the behaviour between the data collections. As such, repeating the data collection during the same day, was a good way to test the reliability of PA item. Yet, after overcoming factors that limit two week gap between surveys, we report this instrument to have moderate agreement. Similar interpretations have been reported in earlier research that did have two to four week gaps between survey administration [17–19].

Problem such as, adolescents’ low ability to recall habitual PA behaviours are common [6, 9, 14]. More specifically, adolescents can experience difficulties to remember their own activities over the last 7 days [33]. In addition, adolescent’s PA often consists of several short bouts [34], can be unplanned, occasional or planned [9], and furthermore, accurate assessment of duration, frequency and intensity of PA can been difficult [35]. These problems can lead to under- [36] or over-estimation of PA [37]. Despite these known pitfalls in self-report of PA, changing the number of categories that are analysed, can influence the proportions that responded the same across consecutive surveys.

We found the best reliability scale was from the two category approach. It should be noted that the two categories were informed by public health measures of adolescents who met or had not met the international PA recommendations [16–18] (participation in at least 60 min of MVPA every day [2]). Therefore, the results reflect upon a monitoring framework based on the international PA recommendations and were considered statistically reliable, rather than dividing the categories at the median for statistical purposes. Creating dichotomous categories has its pitfalls including data loss among adolescents who took part in zero to six days of MVPA, but that would not be informing current methods to monitor PA levels based on the international recommendations. In an earlier study on the validity of adolescents’ PA levels, the cut points that were proposed by Prochaska and colleagues [10] was on five days or more versus zero to four days. A likely reason for that was because the recommendations, at that time, were based on being active on most days [16]. These cut offs may be useful in trend analyses [38], although the item also has scope to be use for monitoring with current recommendation levels. In our study we used the current recommendations as the cut point for the use of two categories [2]. Moreover, after taken into account the cost of administering monitoring surveys, the amount of accuracy has been regarded suitable by other researchers in the past [15, 39] .

Both HBSC and FSPA surveys contained the same single item seven day PA recall item. However in the FSPA survey, there was an item relating light intensity PA on the same screen as the MVPA item. In FPAS, light PA was described in the questionnaire with some example activities, including ‘cycling’. Cycling is an activity that also appears in the description of MVPA. This may have caused confusion for the adolescents responding to the questions as they then tried to calculated how much time they spent doing light PA and MVPA. We suspect, this may have influenced the number of days reported. Due to the importance of monitoring based on the PA recommendations, future surveys should consider the placement of the MVPA item as the first PA item. Moreover, further testing is needed to verify the impact of other PA behavioural intensities in terms of reliability and accuracy of the MVPA measure.

The use of self-reporting in monitoring surveys is fundamentally important when large data sets are combined to provide an overall global surveillance [40]. The use of accelerometers are argued to be more accurate than self-reported questionnaires, especially with young adolescents [15]. However accelerometer measurements have limitations when there it comes to national monitoring surveys, such as, additional expense of devices for large-scale studies [36, 41], logistics in carrying out week long data collection according to the instructions [41], and the possibility still of reported under- and overestimation of PA due to accelerometer type, placement and types of analyses. For example, higher levels of PA than the norm have been reported during the observation period [42]. Underestimation may occur, because accelerometers do not measure some sports like rowing, cycling, have to be removed during water based activities or during contact sports [30, 43]. There are benefits with device based measures as well as with self-reported surveys. In terms of health promotion, it is critical to get the user’s perception of physical activity levels, and that could be obtained through self-reported data.

The HBSC study is a cross-national study where countries are required to use the same protocols to make international reporting feasible. Financial pressures to print surveys, limited access to computers in classrooms, ethical permissions, response biases, wording and question ordering are some of current issues related to the way HBSC data is collected [44]. Participation in the survey is anonymous, which can make it difficult to combine device based data with the HBSC survey. In our study, we used a unique identifying code for each respondent, in the questionnaire to link between the two surveys. The success of this method has paved way for protocol development discussions to use the database unique identifier as a way to link other important data that may be collected, such as device based measures of physical activity.

Survey designers may need to consider an array of considerations such as question order, layout, and number of items on a page or screen to make detailed comparisons in the way adolescents respond to the MVPA questionnaire. Reducing systematic errors in design are important actions for future test-retest studies. As noted in the methods section, the question order and the response methods had subtle differences, and these differences may need to be considered when reviewing the results from this study.

### Limitations

The results of the study are limited to a few conditions. A proportion of pupils who completed the HBSC study, completed the FSPA, and this may have presented further bias to the results. However, we tested the differences in MVPA in the HBSC study between pupils who did and did not complete the FSPA survey, and the differences were not statistically significant. Despite this test, it does not attribute to the way non-responders of FSPA would perform under the test-retest condition. Pupils who were in special education classes or in special schools were not included in the study, limiting the results to be generalizable to the general school setting. Pupils aged 12y and 14y were not included and thus a gap in knowledge between the ages is missing.

## Conclusion

This study was the first assessment of conducting a test-retest reliability of PA measures across two consecutively administered national representative surveys in Finland. The overall findings from this study suggest there was moderate agreement or acceptable reliability of the measure for monitoring purposes. There were differences in the prevalence of daily MPVA due to survey design. However, the self-reported preceding seven-days PA item, when used as a marker for reaching or not reaching international PA recommendations for health, was recommended in boys aged 13- and 15-years old, and girls aged between 11- and 15-years old.

## Abbreviations

FSPA: Finnish School-aged Physical Activity
HBSC: Health Behaviour in School-aged Children
ICC: Intraclass Correlation Coefficient
MVPA: Moderate to vigorous physical activity
PA: Physical Activities
PPS: Probability Proportional to Size
